# Robust Bioinformatics Approaches Result in the First Polygenic Risk Score for BMI in Greek Adults

**DOI:** 10.3390/jpm13020327

**Published:** 2023-02-14

**Authors:** Maria Kafyra, Ioanna Panagiota Kalafati, Maria Dimitriou, Effimia Grigoriou, Alexandros Kokkinos, Loukianos Rallidis, Genovefa Kolovou, Georgios Trovas, Eirini Marouli, Panos Deloukas, Panagiotis Moulos, George V. Dedoussis

**Affiliations:** 1 Department of Nutrition and Dietetics, School of Health Science and Education, Harokopio University, 17671 Athens, Greece; 2 Department of Nutrition and Dietetics, School of Physical Education, Sport Science and Dietetics, University of Thessaly, 42132 Trikala, Greece; 3 Department of Nutritional Science and Dietetics, School of Health Science, University of the Peloponnese, Antikalamos, 24100 Kalamata, Greece; 4 First Department of Propaedeutic and Internal Medicine, Laiko General Hospital, Athens University Medical School, 11527 Athens, Greece; 5 Second Department of Cardiology, Medical School, National and Kapodistrian University of Athens, Attikon Hospital, 12462 Athens, Greece; 6 Cardiometabolic Center, Metropolitan Hospital, 18547 Piraeus, Greece; 7 Laboratory for the Research of Musculoskeletal System “Th. Garofalidis”, School of Medicine, National and Kapodistrian University of Athens, KAT General Hospital, Athinas 10th Str., 14561 Athens, Greece; 8 William Harvey Research Institute, Barts and The London School of Medicine and Dentistry, Queen Mary University of London, London EC1M 6BQ, UK; 9 Institute for Fundamental Biomedical Research, Biomedical Sciences Research Center ‘Alexander Fleming’, 16672 Vari, Greece; 10 Genome Analysis, 17671 Athens, Greece

**Keywords:** polygenic risk score (PRS), bioinformatics, body mass index (BMI), Greek adults

## Abstract

Quantifying the role of genetics via construction of polygenic risk scores (PRSs) is deemed a resourceful tool to enable and promote effective obesity prevention strategies. The present paper proposes a novel methodology for PRS extraction and presents the first PRS for body mass index (BMI) in a Greek population. A novel pipeline for PRS derivation was used to analyze genetic data from a unified database of three cohorts of Greek adults. The pipeline spans various steps of the process, from iterative dataset splitting to training and test partitions, calculation of summary statistics and PRS extraction, up to PRS aggregation and stabilization, achieving higher evaluation metrics. Using data from 2185 participants, implementation of the pipeline enabled consecutive repetitions in splitting training and testing samples and resulted in a 343-single nucleotide polymorphism PRS yielding an R^2^ = 0.3241 (beta = 1.011, *p*-value = 4 × 10^−193^) for BMI. PRS-included variants displayed a variety of associations with known traits (i.e., blood cell count, gut microbiome, lifestyle parameters). The proposed methodology led to creation of the first-ever PRS for BMI in Greek adults and aims at promoting a facilitating approach to reliable PRS development and integration in healthcare practice.

## 1. Introduction

According to WHO estimates for 2016, a considerable 49% and 13% of the global adult population presented overweight or obesity, whereas worldwide obesity prevalence has tripled since 1975 [1]. In this context, respective linear predictions dictate that about 50% of the global population will suffer from obesity by 2030 should similar increasing trends continue uninterrupted [2]. Increased body weight and fat accumulation are evidently directly related to elevated cardiometabolic risk and, subsequently, augmented prevalence of chronic diseases related to glycemic and lipidemic profile, such as type 2 diabetes and cancer [3]. Due to its preventable nature and demand for effective prevention strategies [4], current research focuses on deepening understanding of multifactorial obesity etiology by focusing on the quantified role of genetic predisposition and its reciprocal relation with lifestyle and environmental determinants in populations with various characteristics. 

Indeed, aggregation of multiple single nucleotide polymorphisms (SNPs) in construction of polygenic risk scores (PRS) is increasingly gaining ground as a practical tool to enable quantification and interpretation of genetic information on phenotypic variance. From identification of the first 97 key BMI-related variants [5] up to creation of multiple BMI-specific PRSs presented in the PGS Catalog database [6], using polygenic predictions is increasingly viewed as a useful tool to assess and explain the relevant attributed obesity variance [7,8,9,10,11]. The advantages of the role of PRS use for disease prevention and augmented accuracy in precision medicine are discussed in the context of potentially increasing both personal and clinical utility [12]. Recent studies show that inclusion of PRS in prediction models for certain disease outcomes, such as cardiovascular disease or cancer, carries similar importance to other contributing factors, namely lipidemic biomarkers or smoking [13,14,15]. For that reason, future PRS integration in personalized medicine is deemed useful for disease diagnosis, risk prediction and forming contextualized lifestyle recommendations [13]. 

The current literature highlights the need for an efficient translational approach to integrating PRS use into daily practice, potentially via inclusion in tools predicting disease risk [13]. In an effort to increase validity and straightforward application, various methodologies for PRS creation have been suggested. In the case of examining BMI, such examples refer to conduct of large genome-wide association studies (GWAS) and subsequent inclusion of significant SNPs in the form of a score [11,16], a priori aggregation of literature-based SNPs [9] or even use of other techniques, such as functional data analysis [17]. However, most approaches suggested to date focus on the use of one methodology and do not display increased portability and applicability across populations [18]. The need of improving their constructive parameters is, therefore, deemed central in order to increase PRS validity and wider implementation [12].

Hereby, we introduce the use of a novel, automated and iterative approach for PRS construction using repetitive sample splitting processes, informed decision-making through real-time comparison of different summary statistics’ methodologies and aggregation of PRS candidates based on a stabilizing iterative procedure. We present the results of its application in creating the first PRS for BMI in Greek adults using data from a unified database of three separate cohorts. The suggested outlined pipeline constitutes an innovative approach in facilitating PRS construction in a straightforward manner, applicable to cohorts of various sizes and characteristics. 

## 2. Materials and Methods

### 2.1. Study Population

For the purpose of the present analyses, data from three cohorts of Greek adults were used, namely the case-control Greek Non-Alcoholic Fatty Liver Disease (NAFLD) study [19], the cross-sectional OSTEOS study [20] and the case-control THISEAS (The Hellenic Study of Interactions between Single Nucleotide Polymorphisms and Eating in Atherosclerosis Susceptibility) [21] study. All studies were approved by the Research Ethics Committee of Harokopio University of Athens and further required participants’ written informed consent prior to enrolment (NALFD protocol number: 38074/13-07-2012, OSTEOS protocol number: 15/8-12-2005, 8/12/2005, THISEAS protocol number: 10/9-6-2004, 14/6/2004). 

The detailed protocols of all three studies have been previously described elsewhere [19,20,21,22,23]. Briefly, the NAFLD study recruited adult participants without liver disease/injury and reporting absence of excess alcohol drinking at the time of induction to the study. Volunteers were recruited from the Outpatient Clinics of the First Department of Propaedeutic and Internal Medicine in Laiko General Hospital, during the period 2012 to 2015 [19]. Recruits were further screened for NAFLD through abdominal ultrasound and deemed as controls in the absence of hepatic steatosis or in the presence of mild-stage, or cases in presence of moderate or severe hepatic steatosis [20]. Concerning the nodes of the OSTEOS study, 970 community-dwelling adults were recruited from rural and urban areas of Greece and assessed for quantitative ultrasound (QUS) parameters of bone health during the 2010–2012 period and in cooperation with the Hellenic Society for the Support of Patients with Osteoporosis and the Laboratory for the Research of Musculoskeletal System “Th. Garofalidis”, School of Medicine, National and Kapodistrian University of Athens [21]. Last, within the THISEAS study, a total of 2565 participants were recruited from three Athenian hospitals, open protection centers and municipalities during the years 2006–2010. Recruits were mainly assessed using coronary angiography information and were categorized as controls if they presented negative coronary findings or a negative stress test or did not report any related clinical symptoms. Volunteers were categorized as cases in the presence of acute coronary syndrome or stable coronary artery disease (> 50% stenosis in ≥ 1/3 main coronary vessels) [22,23]. 

### 2.2. Anthropometric Measurements

Anthropometric characteristics, including body weight and body height, were measured for all three studies. Body weight was measured using the TANITA Segmental Body Composition Analyzer BC-418 and a calibrated scale to the nearest 0.1 kg. Height was calculated to the nearest 0.5 cm using a mounted stadiometer. Participants were barefoot and maintained light clothing and measurements occurred twice and average values were kept as final in all projects. All measurements were conducted by trained professionals. BMI was calculated for all participants via use of the following formula:

$$\mathrm{BMI}\left(\frac{\mathrm{kg}}{m^{2}}\right)=\mathrm{Body}\;\mathrm{Weight}\left(\mathrm{kg}\right)/\;{\left(\mathrm{Body}\;\mathrm{Height}\right)}^{2}\left(m^{2}\right)$$

Participants in all studies were classified based on BMI values in the categories of underweight (BMI < 18.5 kg/m^2^), normal weight (18 kg/m^2^ ≤ BMI < 25 kg/m^2^), overweight (25 kg/m^2^ ≤ BMI < 30 kg/m^2^) or obese (BMI ≥ 30 kg/m^2^). Within-study group differences in BMI were calculated using Kruskal–Wallis tests.

### 2.3. Genotyping Analyses

For the NAFLD study, DNA samples were isolated using peripheral blood lymphocytes and genotyped via use of the Infinium CoreExome-24 BeadChip, Illumina genome-wide SNP array (with 567,218 fixed markers). OSTEOS’ DNA samples were isolated from buffy coats and genotyped using the Axiom Precision Medicine Diversity Research Array [with over 850,000 SNPs, insertions, deletions and copy number variations (CNVs)]. DNA samples from the THISEAS study were extracted from whole blood and genotyped using the Illumina Metabochip (with about 200.000 SNPs). 

### 2.4. Preprocessing and Statistical Analysis

#### 2.4.1. Dataset Merging and Genotype Imputation

Prior to joint statistical analysis and PRS derivation, the phenotypic and genotypic data of the three populations were merged. While the phenotypic integration was straightforward and comprised the simple join of the common phenotypes across the three datasets, the following steps were followed for the genotypic data which were converted to PLINK [24] 1.9 BED+BIM+FAM filesets. First, the PLINK filesets from NAFLD and THISEAS were imported into R version 4.2.0. using facilities from the package snpStats, version 1.46.0. Then, the process of merged dataset creation started with identifying the identical SNPs between the two datasets in terms of accession numbers, position and alleles. For the common but non-identical SNPs in terms of alleles, it was checked whether they could be resolved with strand-flipping. Those SNPs that could not be resolved with strand-flipping were not pointing to the same risk allele. This was resolved by querying online resources (Ensembl with the R package biomaRt, version 2.52.0 and dbSNP with the R package rsnps, version 0.5.0). After the resolution, samples where the risk allele was changed based on online search were subjected to allele switching to maintain proper risk allele copies in the merged dataset. SNPs for which alleles could not be resolved by any means were dropped from the merged dataset. Finally, the SNPs and genotypes unique to each dataset were appended to the common ones to form the final SNP set. The same appending was applied to the samples of each dataset.

As expected, the aforementioned process created many missing genotypes, especially regarding non-common SNPs between the two datasets. To impute them, an iterative imputation approach was followed using facilities from package snpStats. The package includes genotype imputation functions based on linear regression of neighboring SNPs. This process was repeated until no further genotype imputation was possible. For the remaining missing genotypes of the merged dataset, a k-nearest-neighbors-based imputation technique was applied, implemented in the R package scrime, version 1.3.5.

The merging and the imputation process resulted in a merged NAFLD–THISEAS dataset. The OSTEOS dataset was merged with the latter by repeating all the aforementioned steps, resulting in a merged NAFLD–THISEAS-OSTEOS dataset. The final merged dataset was exported to PLINK format using functions from the snpStats package. Next, to enhance the pool of SNPs for PRS derivation, the merged dataset was extended using IMPUTE2 software [25] using the bundled 1000 Genomes Project reference panel. The imputed and extended dataset was re-imported to R for further analysis. 

#### 2.4.2. Data Filtering and Summary Statistics

The first filter applied to genotypic data was to exclude poorly imputed genotypes; therefore, SNPs with an IMPUTE2 INFO score less than 0.9 were excluded. Additional genotype and sample filtering was performed using functionalities from the snpStats package. Specifically, SNPs with an SNP call rate < 95% and minor allele frequency (MAF) < 5% and samples with a sample call rate < 90% were excluded from further analysis. The resulting filtered dataset was further subjected to a second round of genotype filtering based on the Hardy–Weinberg (HWE) equilibrium, where SNPs with HWE *p*-value < 10^−9^ were also excluded from further analysis.

After dataset filtering, principal component analysis (PCA) was performed to capture any underlying population stratification not reflected by the confounders used in the subsequent association tests using R package SNPRelate, version 1.30.1. Subsequently, regression models were fitted for each SNP against BMI phenotype using sex, age, NAFLD case/control and cardiovascular disease status along with selected PCs as correction covariates with the purpose of deriving summary statistics for each SNP, namely effects and statistical significance for contribution of each single SNP to the phenotype. The number of PCs was automatically selected using the Tracy–Widom statistic for assessment of the most significant PCs based on their eigen values [26]. Four different algorithms were used for derivation of summary statistics, namely simple General Linear Models (GLM, R version 4.2.0), statgenGWAS version 1.0.8. [27], SNPTEST version 2.5.4 [28] and PLINK.

#### 2.4.3. Derivation of PRS

Several PRS candidates were derived using PRSice2 [29] combined with an iterative process for PRS derivation and validation and based on the merged dataset from the three populations. The PRS was calculated with the default PRSice2 option, which is:

$$\mathrm{PRS}=\overset{k}{\underset{i=1}{\sum }}\frac{\beta_{i}G_{i}}{Ν}$$

where βi represents the effect of PRS SNP i, Gi is the genotype coding (0, 1, 2 following PLINK notation, for the number of copies of risk alleles) and N the number of samples in the population. The PRS is reported in the figures of the present articles after applying min–max normalization to scale it to values between 0 and 1.

In each iteration, the following actions were performed: first, the total dataset was split to a training set (source set, 80% of samples) and a testing set (target set, 20% of samples). Then, the source set was used to perform de novo association tests for each SNP with four different methods (GLM, statgenGWAS, SNPTEST, PLINK) against the BMI phenotype. Sex, age, NAFLD status and several automatically selected PCs (varying between 5–12 across multiple iterations), using the Tracy–Widom test, were used as confounders in the regression models underlying each of the four methods, resulting in sets of summary statistics derived with each method. Then, these summary statistics were used along with the target dataset as inputs to PRSice2 for extraction of the optimal number of SNPs that would comprise a candidate PRS for the specific iteration. The aforementioned steps, from data splitting up to PRS synthesis with PRSice2, were repeated 100 times. At each iteration, several performance metrics were collected, among which the statistical significance of the PRS and the percentage of additional variance explained by the PRS (R^2^) as returned by PRSice2. At this point, it should be noted that the PRSice2 PRS R^2^ is the difference between the R2 of the “full” model, i.e., a regression model including all the covariates/confounders and the PRS, and the “null” or “reduced” model, i.e., a regression model only with the other covariates without the PRS. The PRS R^2^ values were collected for each iteration, resulting in a baseline distribution that would be used later for assessing the statistical significance of the final PRS. 

After completion of PRS derivation iterations, SNPs comprising PRS candidates for each summary statistics method were aggregated and number of appearances (frequency) of each SNP in the 100 iterations was counted considering an SNP to be appearing at least 5 times in order to further proceed to the downstream procedures. Then, for each frequency, a PRS comprising the SNPs appearing equally or above this frequency was assembled with effects averaged over iterations where each SNP appears and evaluated using previously described source/target dataset splits and linear regression, resulting in a series of evaluation metrics, among which also the PRS R2 as described above. This was repeated for all observed frequencies and a distribution of PRS R2 values was created. The PRS R^2^ values were further penalized based on number of SNPs in PRS according to the following formula:

$$R_{P}^{2}=\sqrt{\frac{R_{\mathrm{PRS}}^{2}}{\log\left(N\right)}}$$

where $R_{P}^{2}$ is the PRS R^2^ and N is the number of SNPs in the PRS. Then, a set of pre-final PRS candidates was defined by detecting local maxima in the $R_{P}^{2}$ distribution, reflecting PRSs with high values of $R_{P}^{2}$. The final PRS was selected based on the highest $R_{P}^{2}$ value. The statistical significance of the aggregated PRS R^2^ as well as the $R_{P}^{2}$ was assessed using an empirical bootstrap defined as number of times where the baseline PRS R^2^ was greater than the aggregated PRS R^2^ divided by number of iterations.

## 3. Results

### 3.1. Population Characteristics 

The anthropometric characteristics of the unified sample are described in Table 1. Overall, we used available data from 2083 participants, namely 342 participants from the NAFLD study, as well as 791 and 950 participants from the OSTEOS and THISEAS studies, respectively. A total of 841 men and 1242 women were included, with a median age of 53 years (calculated at 2075 participants) and a median BMI of 27.38 kg/m^2^. Within the respective databases, participants presented median BMIs in the spectrum of overweight for all three studies (NAFLD median BMI = 26.5 kg/m^2^, OSTEOS median BMI = 26.91 kg/m^2^ and THISEAS median BMI = 27.81 kg/m^2^). BMI was not statistically significantly different between the NAFLD and OSTEOS studies but did present a statistically significant difference between the NAFLD and THISEAS as well as the OSTEOS and THISEAS studies (*p* < 0.001 for both pairs). Differences in age were also statistically significant between all studies (*p* < 0.001 for the Kruskal–Wallis test).

Differences in BMI levels across the two sexes were statistically significant in the overall sample (*p*-value < 2.2 × 10^−16^), with men presenting higher values. Among the overall sample, 614 participants presented BMI in the range of 18.5–24.99 kg/m^2^ (31.43% men, 68.56% women), whereas 875 and 579 participants presented overweight and obesity, respectively (Table 2). Most participants presenting overweight or obesity were in the THISEAS study (n = 730). 

Regarding genotypic data, after imputation of IMPUTE2 with data from 1000 genomes project as a reference panel, a total of 24,307,245 variations were made available. Subsequently, variants with imputation confidence (INFO score returned by IMPUTE2) less than 0.9, structural and copy-number variations were excluded from further analysis. All downstream analyses were based only on known variants (i.e., variants recorded in dbSNP). This process led to 1,454,104 variants interrogated for PRS candidates. With respect to samples, 1970 (94.6%) had complete phenotypic records for covariates interrogated in regression models and included in further analyses.

### 3.2. Summary Statistics for PRS Derivation

Summary statistics for the merged dataset were calculated with BMI phenotype as a response variable and using the extended (imputed based on the 1000 genomes external reference panel) and further filtered genotypic dataset. In order to properly estimate the effects of individual SNPs that potentially contributed to the BMI phenotype in the unified dataset, we applied four different frameworks for summary statistics estimation, namely a simple generalized linear model (GLM) as implemented in the R statistical language, the regression algorithm implemented in the R package statgen GWAS as well as the SNPTEST software and the more generalized PLINK framework. In all cases, the sex, age, NAFLD status and cardiovascular disease status of individuals were incorporated in the regression models as confounders, along with several automatically selected principal components to capture potential underlying population stratifications not reflected by the other confounders. The four sets of summary statistics were used as input to PRSice2 along with the target samples in an iterative PRS derivation procedure, as described in Materials and Methods. To evaluate the performance of each summary statistics estimation method, we used the PRS R^2^ metric returned by PRSice2, which measures percentage of BMI variability explained by the PRS in the regression models. The PRS R^2^ values for each method were averaged over 100 PRS derivation iterations (Supplementary Figure S1) and the method that yielded the highest PRS R2 was selected to provide the summary statistics for final PRS derivation. In our case, SNPTEST yielded the highest average PRS R^2^ (0.012 ± 0.006, pmin = 0.0002, pmedian = 0.0375, pmax = 0.3194), followed by GLM (0.011 ± 0.006, pmin = 0.0003, pmedian = 0.0697, pmax = 0.4251) and statgenGWAS (0.010 ± 0.006, pmin = 0.0005, pmedian = 0.0718, pmax = 0.3579). PLINK yielded the lowest average PRS R^2^ values but with the smallest variability across 100 iterations (0.009 ± 0.004, pmin = 0.0002, pmedian = 0.0802, pmax = 0.5282).

### 3.3. Selection of a PRS

After completion of 100 PRS derivation iterations, we assessed the stability of the extracted PRSs (Supplementary Figure S2). We observed that, in our case, PRS extraction process was highly dependent on source (training) dataset summary statistics. As a result, the SNP content of each PRS greatly varied between iterations, therefore affecting the performance of the latter and its contribution in explaining BMI. In order to mitigate the observed PRS instability, the 100 different SNP sets comprising the 100 different PRSs returned by PRSice2 with SNPTEST summary statistics were aggregated (Supplementary Table S1) as described in Materials and Methods, requiring that an SNP considered for inclusion in a PRS candidate should appear at least five times in the end of the iterative procedure.

**Figure 1:**
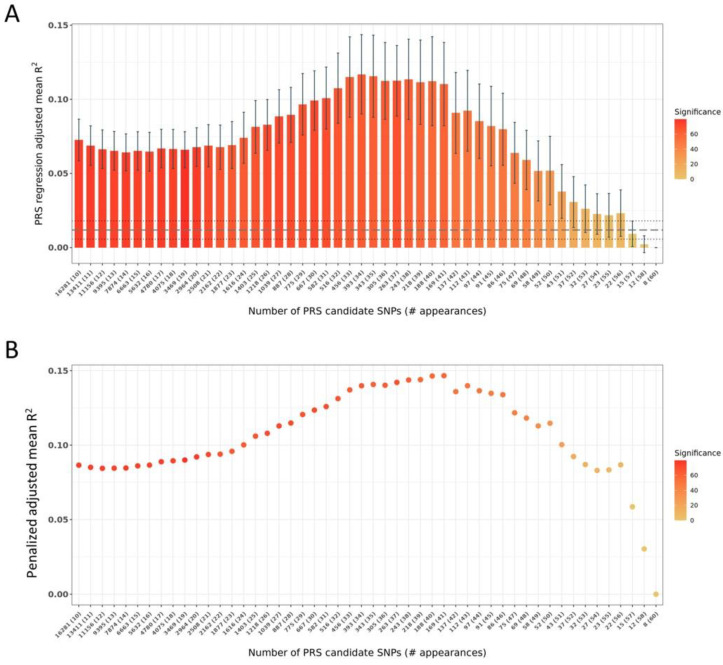
Mean PRS and penalized PRS R^2^ for the assembled PRS candidates based on their frequency of appearance over 10 iterations as described in Materials and Methods. (**A**). Mean PRS R2 +/− standard deviation for PRS candidates assembled from SNPs at different frequencies of appearance in the PRS candidates across 100 PRS extraction iterations. The vertical axis depicts the mean adjusted PRS R^2^, while the horizontal axis depicts the number of SNPs in each PRS candidate. The number inside the parentheses next to the number of SNPs in the horizontal axis depicts the SNP frequency of appearance in the PRS. For example, 393 (34) means that the PRS at that particular R^2^ consists of 393 SNPs that appear at least 34 times over 100 iterations. The color scale denotes the statistical significance (Student’s *t*-test *p*-value in −log10 scale) of the adjusted R^2^ distribution over 100 de novo PRS extraction iterations (baseline R^2^) as compared to the adjusted R2 distribution of each assembled PRS candidate in the horizontal axis. The mean baseline (derived directly from PRSice2 outcomes for each iteration) R2 is depicted with the dashed grey horizontal line, and the dotted grey horizontal lines depict the standard deviation of the former. (**B**). Mean penalized according to the number of SNPs PRS R^2^.

Subsequently, several PRS candidates were assembled with SNP content based on frequency of appearance of the latter across the aggregated SNP set, new regression models were created based on the initial target dataset splits used by PRSice2 and PRS R^2^ values were assembled (Figure 1A) along with their respective significance when compared with the baseline PRSice2 PRS R^2^ distribution. As our goals included derivation of a PRS with a less extended number of SNPs but of high predictive value as a PRS with a larger number of SNPs, the new PRS R^2^ values were further penalized based on the number of SNPs that each PRS candidate included (Figure 1B). Then, using the resulting distribution of penalized PRS R^2^ values, we detected local maxima, denoting both high predictive value and lower SNP content. The number of SNPs yielding an adequately high penalized PRS R^2^ while maintaining significance when compared to the baseline PRS R^2^ distribution was found to be 343 (PRS R^2^ = 0.1156 ± 0.0277). Notably, our iterative and aggregative PRS derivation process resulted in a PRS with ~10 times improved explanatory power (bootstrap *p*-value = 0, Figure 1A) than using PRSice2 alone. 

### 3.4. PRS Evaluation

Next, we further evaluated the final 343-SNPs-selected PRS for BMI using the total merged dataset coupled with an iterative 10-fold cross-validation process, where, in each iteration of the process, we left out 5–50% of the total dataset samples, each time increasing the left-out samples by 5% and creating regression models including (full) and excluding (reduced) the PRS while maintaining the other covariates (Supplementary Table S2). Overall, the PRS increased the predictive power of the models by 31–33%, with the minimum PRS R^2^ value observed at 0.3159 ± 0.0190 (*p*-value = 4 × 10^−87^) when leaving out 50 of samples, with the maximum value at 0.3279 ± 0.0114 (*p*-value = 9 × 10^−130^). A final regression model using the 343-SNP PRS for BMI with the total merged dataset yielded a PRS R^2^ = 0.3241 (beta = 1.011, *p*-value = 4 × 10^−193^). Finally, to evaluate the ability of the 343-SNP PRS to characterize close phenotypes, we created a regression model with the same covariates but using population weight instead of BMI. The model yielded PRS R^2^ = 0.2313 (beta = 2.702, *p*-value = 4.15 × 10^−158^, Supplementary Figure S3).

### 3.5. PRS for BMI

**Figure 2:**
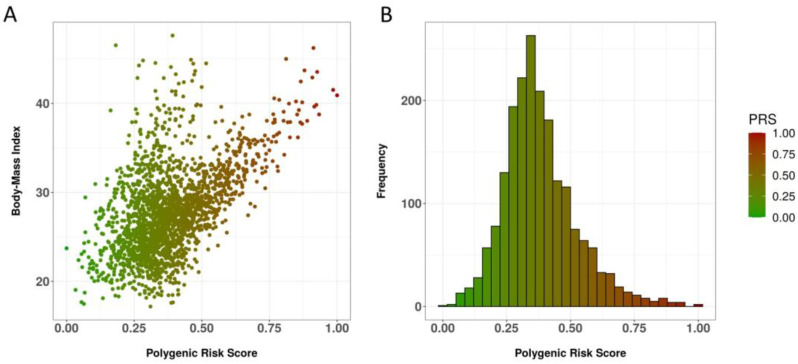
Correlation of the 343-SNP PRS for BMI with the phenotype and PRS distribution. (**A**). The BMI phenotype across the merged dataset is plotted against the min–max-normalized PRS value for each individual. (**B**). Histogram depicting the min–max-normalized PRS distribution for all individuals in the merged dataset.

The aforementioned 343-SNP PRS deriving from using SNPTEST displayed a statistically significant association for BMI (beta = 1.011, *p*-value = 4 × 10^−193^) and a positive correlation, where increased PRS values were associated with increased BMI levels. As shown in Figure 2, the examined population presented an overall median risk, with most observations met in the 0.25–0.50 range. Out of the 343 SNPs identified in the PRS (see Supplementary Table S3), automatically identified known associations included in the GWAS Catalog were displayed for 16 SNPs, namely rs2710804 (27 associations) and rs2955742 (five associations) (see Table 3). 

## 4. Discussion

The present study sought to investigate application of an automated pipeline for PRS extraction using data from the three Greek studies of NAFLD, OSTEOS and THISEAS. In this population of Greek adults, the constructed PRS displayed a statistically significant association for BMI, with an R^2^ of 0.3241 (beta = 1.011, *p*-value = 4 × 10^−193^). The iterative pipeline presented here attempts to address various matters on PRS extraction, namely selection of an appropriate threshold for SNP inclusion and prediction accuracy [18] as well as stability of the SNP content of PRS candidates across different training and test dataset splits.

In attempting to strengthen PRS construction methodology [30], this pipeline proposes implementation of iterative processes through repetitive steps of sample splitting, aggregating SNP frequency and effect size as well as comparative use of summary statistic metrics and consideration of lifestyle and genetic covariates. As a result, the suggested PRS includes a less extended number of variants but of high explanatory power. In this spectrum, this effort aims at facilitating construction of high-validity PRSs and subsequently promoting their use as a diagnostic tool accounting for various individual characteristics in daily practice. Use of the information of increased or reduced genetic risk for elevated BMI values, as demonstrated by the PRS, can potentially be translated in clinical practice to intensify (in the case of increased risk) or modify and personalize recommendations on lifestyle parameters to combat overweight and obesity. 

To the best of our knowledge, the present study constitutes the first attempt to develop a PRS for BMI using data from a Greek population and a previous attempt for construction of a PRS has only been referred to once before in the current literature, exploring Parkinson’s disease in older Greek adults [31]. Implementation of the suggested aggregated methodology refers, among others, to (a) repetitive splitting of the overall sample; (b) comparative use of different summary statistics in an attempt to reduce population size and SNP selection bias, respectively. Thus, future work will concern attempts in replicating the proposed PRS in wider populations of different ancestry. 

Other attempts to create PRSs for BMI in populations of European ancestry are extensively described in the current literature, with an overall number of 56 BMI-related entries in the PGS Catalog [6]. All referred entries include parts of populations of European ancestry but present a wide range in the numbers of PRS-included variants, from a few tens up to several thousand or millions, with these numbers possibly limiting their effective usage in research or clinical settings. Although the PRS proposed here includes only 343 SNPs, the yielded R^2^ of 0.3241 is substantially comparable, and, in some cases, higher, than the ones presented in other PRSs from BMI, which include thousands of SNPs [6]. An overall advantage is also observed when comparing the present results to other attempts in European populations, which have a priori calculated the effect of literature-based PRSs using a limited amount of SNPs. Use of our proposed pipeline is an advanced tool due to the notion that the aggregated approach of splitting processes strengthens identification of appropriate and sometimes novel SNPs increases the validity of the results and makes up for the need to have a very large sample size.

In the current study, we observe links for various indices related to cardiovascular profile for twelve out of the sixteen variants with GWAS-Catalog-identified associations. The latter could be explained by inclusion of data for THISEAS participants with diagnosed cardiovascular disease (19.58% of the participants). Although the mediating effect of BMI is usually accounted for when investigating the effect of genetic or polygenic risk scores on indices of cardiovascular disease, the reciprocal relation between variation in cardiometabolic indices levels and BMI levels has not been extensively demonstrated through BMI-PRS-included, CVD-related variants. Out of the associated SNPs, the C allele of the rs2710804-included variant presents the majority of reported associations, namely with cell count types (platelets, leukocytes, lymphocytes) and even measurements of C-reactive protein. In this context, the negative effect of the T allele observed in our study (β = −0.1356) could denote a positive relation of the C allele with metabolic pathways of inflammation and disturbed immunological responses in the subsequent increasing effect of BMI values. 

Interestingly and among this PRS’s novel associations, we find two variants previously linked to gut microbiome measurements in populations of European ancestry. More specifically, Rühlemann MC et al. previously associated the rs480039 SNP with a 0.082571946 unit increase in P_Bacteroidetes abundance among German individuals [32]. Similarly, a 0.1019 unit increase in the abundance of parabacteroides in stools of individuals of Finnish ancestry for the A allele of the rs12673506 SNP was shown by Qin et al. [33]. Comparably, our study showed that the G allele of the rs480039 and rs12673506 variants was negatively related to BMI levels (β = −0.1736 and β = −0.1850, respectively). This is not the first time that the *Parabacteroides* genus has been linked to body weight. The majority of studies denote a higher *Firmicutes:Bacteroidetes* ratio and a generalized reduction in species variation in individuals with increased body weight or obesity [34], and different studies have found positive associations between genus and normal weight or weight loss in mice, as well as fat loss in humans [35,36,37,38,39]. It is plausible that the corresponding SNPs are further linked to BMI through the genus’s role in gut production of bile acids and succinate, which have, in turn, been associated with reduction in body weight [38]. 

When referring to SNPs related to lifestyle, our suggested PRS included one variant related to well-being (variant rs17662327) and one variant associated with exercise (rs10252228). More specifically, in our sample, presence of the T allele of the former SNP was linked to a 0.1471 change in BMI levels. Previously, Okbay et al. demonstrated a 0.0182 unit increase in sentiment of life satisfaction or emotional well-being of adults for the T allele [39]. Our study further showed that presence of the A allele of the rs10252228 SNP was related to higher BMI values (β = 0.1206). This finding could be in accordance with the 0.027 unit increase in exercise associated with leisure time shown for the SNP’s G allele in Japanese adults [40], meaning that the positive effect of the A allele on BMI could be mediated by individuals’ low exercise levels. 

One of the great strengths of the present study entails implementation of our novel methodology for extraction of PRS, which enables effective management and analysis of the vast amounts of genetic data required for such analyses. The automated pipeline enables practical application of our suggested holistic approach for extensive examination of thousands of SNPs, leading to identification of various novel associations. Through the methodological approach of applying a repetitive process of continuous adjustment of the R^2^ measure for the number of each-time-associated SNPs, the pipeline aims to facilitate integration of PRS use in daily healthcare practice, for example as part of widely distributed consumer reports. It should be stressed that, as this methodology is based on the highest R^2^ values of the aggregate PRS candidates, it ensures high explanatory power of the reduced signature. At the same time, it mitigates any computational and data management burden imposed by PRSs with large (up to millions) numbers of SNPs. 

Limitations of the present study mainly concern power given the restrained participant sample size available for conducting analyses. Another limitation refers to use of a unified database of participants from three different studies. It is possible that variation in participant characteristics and bias accompanying use of a large analogic sample size of participants with cardiovascular disease played a considerable part in identifying associations between BMI and SNPs related to regulation of cardiovascular indices. However, we determined that much of the potential variability introduced by the fact of joining three databases was successfully captured by one of the PCs incorporated in the model. In addition, although the hypothesized pathways through which the identified SNPs potentially affect BMI levels provide insight for novel relations, there is little evidence to establish direct causal relationships. However, the present analysis sets a foundation for the suggested causal SNPs, and further research is also needed to explore the possibility of relations through their role as proxies for different associated variants. 

## 5. Conclusions

The present paper describes creation of the first PRS for BMI in Greek adults by introducing use of a novel, automated pipeline for PRS extraction. The findings of this study lead to identification of several novel SNPs associated with BMI, potentially through their implication in various metabolic pathways related to traits of cardiometabolic profile and gut microbiome. Our data provide novel insights into interactions of various biological pathways implicated in formation of BMI levels and subsequently affecting its individual variation across different populations. The suggested pipeline aims at promoting maximization of PRS integration in daily healthcare practice by enabling rapid and straightforward development of risk scores. In this regard, this first-ever PRS of a Greek population highlights the need for further development of PRSs for anthropometric traits in larger databases of Greek adults and sets a foundation for wider use of the described iterative PRS methodology.

## Figures and Tables

**Table 1:** Descriptive characteristics of the NAFLD, OSTEOS and THISEAS study populations.

	All			NAFLD			OSTEOS			THISEAS		
	All (n = 2075 for age, n = 2083 for BMI)	Men (n = 841)	Women (n = 1234 for age, n = 1242 for BMI)	All (n = 342)	Men (n = 140)	Women (n = 202)	All (n = 783 for age, n = 791 for BMI)	Men (n = 101)	Women (n = 682 for age, n = 690 for BMI)	All (n = 950)	Men (n = 600)	Women (n = 350)
	Med (IQR)											
Age	53 (18)	54 (19)	52 (19)	47 (18)	44 (17)	50 (16)	50 (18)	47 (28.5)	51 (16.25)	59 (19)	58 (18.75)	60 (21)
BMI (kg/m^2^)	27.38 (6.18)	27.68 (5.34)	27.02 (7.10)	26.5 (6.23)	26.8 (4.54)	25.9 (6.98)	26.91 (6.81)	26.70 (5.13)	26.94 7.01)	27.81 (5.80)	27.88 (5.43)	27.77 (6.51)

BMI: body mass index, Med: median, IQR: interquartile range.

**Table 2:** Frequencies of BMI categories across the three studies.

	BMI < 18.5 kg/m^2^			18.5 kg/m^2^ ≤ BMI < 25 kg/m^2^			25 kg/m^2^ ≤ BMI < 30 kg/m^2^			BMI ≥ 30 kg/m^2^		
	All	Men	Women	All	Men	Women	All	Men	Women	All	Men	Women
All	15	0	15	614	193	421	875	405	470	579	243	336
NAFLD	3	0	3	117	36	81	141	74	67	81	30	51
OSTEOS	10	0	10	279	34	245	300	43	257	202	24	178
THISEAS	2	0	2	218	123	95	434	288	146	296	189	107

BMI: body mass index.

**Table 3:** List of PRS SNPs with known associated traits in GWAS Catalog.

Consortial Summary Statistics (GWAS Catalog)					Known Associated Traits		Unified Cohort Summary Statistics	
SNP	Nearest gene	Position (Chr:bp)	Alleles	MAF	Effect Allele	Associated Traits	Effect allele	Beta ^1^
rs11668205	IZUMO4	19:2096429-2099593	G/A	0.09 (A)	N/A	Abnormality of chromosome segregation	G	−0.32575
rs488248	LOC728192	13:105944370	C/A/T	0.23 (C)	T	Response to docetaxel, antineoplastic agent	C	−0.17048
rs480039	SLC35F3	1:234290732	G/A/C/T	0.37 (A)	N/A	Gut microbiome measurement	G	−0.17361
rs2288061	RPL18P13	16:76135833	G/A/C	0.34 (A)	G	Delta-5 desaturase measurement	G	−0.17776
rs2807854	HLX-AS1	1:220856499	T/C/G	0.25 (T)	T	LDL, apoB measurements	T	−0.13816
rs2955742	TMEM266	15:76153791	G/A	0.10 (A)	A	Serum urea, cystatin c, creatinine, urate, glomerular filtration measurement	G	−0.19108
Rs2710804	SEPT7,EEPD1	7:36044919	T/C	0.23 (C)	#N/A	Fibrinogen measurement	T	−0.1356
rs2710804	N/A	7:36044919	T/C	0.23 (C)	C	Serum alanine aminotransferase measurement	T	−0.1356
rs2710804	N/A	7:36044919	T/C	0.23 (C)	C	Lymphocyte count	T	−0.1356
rs2710804	N/A	7:36044919	T/C	0.23 (C)	C	Platelet count	T	−0.1356
rs2710804	N/A	7:36044919	T/C	0.23 (C)	C	Lymphocyte count	T	−0.1356
rs2710804	KIAA1706	7:36044919	T/C	0.23 (C)	C	C-reactive protein measurement	T	−0.1356
rs2710804	AC083864.3	7:36044919	T/C	0.23 (C)	C	Leukocyte count	T	−0.1356
rs2710804	N/A	7:36044919	T/C	0.23 (C)	C	Neutrophil count	T	−0.1356
rs2710804	N/A	7:36044919	T/C	0.23 (C)	C	Myeloid white cell count	T	−0.1356
rs2710804	N/A	7:36044919	T/C	0.23 (C)	N/A	Leukocyte count	T	−0.1356
rs2710804	SEPT7, EEPD1	7:36044919	T/C	0.23 (C)	N/A	Fibrinogen measurement	T	−0.1356
rs2710804	N/A	7:36044919	T/C	0.23 (C)	C	Lymphocyte count	T	−0.1356
rs2710804	N/A	7:36044919	T/C	0.23 (C)	C	Platelet count	T	−0.1356
rs2710804	N/A	7:36044919	T/C	0.23 (C)	T	Platelet count	T	−0.1356
rs2710804	N/A	7:36044919	T/C	0.23 (C)	C	Leukocyte count	T	−0.1356
rs2710804	AC083864.3	7:36044919	T/C	0.23 (C)	C	Neutrophil count	T	−0.1356
rs2710804	N/A	7:36044919	T/C	0.23 (C)	C	Serum albumin measurement	T	−0.1356
rs2710804	N/A	7:36044919	T/C	0.23 (C)	C	C-reactive protein measurement	T	−0.1356
rs2710804	EEPD1	7:36044919	T/C	0.23 (C)	C	Fibrinogen measurement	T	−0.1356
rs2710804	N/A	7:36044919	T/C	0.23 (C)	C	Neutrophil count	T	−0.1356
rs2710804	LOC101928618	7:36044919	T/C	0.23 (C)	T	Serum alanine aminotransferase measurement	T	−0.1356
rs2710804	N/A	7:36044919	T/C	0.23 (C)	C	Myeloid white cell count	T	−0.1356
rs2710804	N/A	7:36044919	T/C	0.23 (C)	C	Platelet count	T	−0.1356
rs2710804	AC083864.3	7:36044919	T/C	0.23 (C)	C	Lymphocyte count	T	−0.1356
rs2710804	AC083864.3	7:36044919	T/C	0.23 (C)	C	Platelet count	T	−0.1356
rs2710804	AC083864.3	7:36044919	T/C	0.23 (C)	C	Platelet crit	T	−0.1356
rs2710804	N/A	7:36044919	T/C	0.23 (C)	C	Neutrophil count	T	−0.1356
rs2251188	ZNF12, ZNF316	7:6664701	A/C/G/T	0.16 (A)	G	Basophil count, neutrophil count	A	0.13807
rs7589592	ENSG00000237720	2:2709171	T/A/C	0.41 (C)	N/A	Diffuse plaque measurement	T	0.11391
rs1010304	CHD6, EMILIN3	20:41473007	A/G	0.30 (G)	A	Memory performance, word list delayed recall measurement	A	−0.28657
rs12673506	CHN2	7:29382170	G/A	0.24 (A)	A	Gut microbiome measurement	G	−0.185
rs17662327	HNRNPA1P41,JAK2	9:4967587	T/C/G	0.16 (C)	T	Wellbeing measurement	T	0.14714
rs2485662	MEX3A/LMNA	1:156113677	T/C	0.31 (T)	N/A	Triacylglycerol 48:1, triacylglycerol 50:2 measurements	T	0.11601
rs4718965	AUTS2	7:70575462	C/A/T	0.08 (C)	C	Cortical surface area measurement	C	0.19049
rs9847987	intergenic/CFAP20DC-DT	3:59432807	C/T	0.20 (T)	T	Neuritic plaque measurement	C	0.26274
rs10252228	DPY19L1, NPSR1	7:34900427	A/G	0.29 (G)	G	Exercise	A	0.12063

SNP: single nucleotide polymorphism, Chr: chromosome, bp: base pairs, MAF: minor allele frequency, beta: effect size for BMI. ^1^ Results were derived via linear regressions after adjusting for sex, age, NAFLD status and number automatically selected PCs for population stratifications. Effect sizes (betas) and ORs shown for the corresponding SNP and effect sizes (betas) are reported for the respective effect allele.

## Data Availability

Summary statistics and data used for the purposes of the present study are available upon request from the corresponding author. Participant data are not publicly available due to participants’ privacy and ethical restrictions.

## Supplementary Materials

The following supporting information can be downloaded at: https://www.mdpi.com/article/10.3390/jpm13020327/s1, Figure S1: Mean PRSice2 PRS R^2^ +/− standard deviation for each performed summary statistics derivation method across 100 PRS extraction iterations; Figure S2: Stability of the PRS candidates over 100 PRS extraction iterations as described in the main text; Figure S3: Correlation of the 343-SNP PRS for BMI with the weight phenotype and PRS distribution; Table S1: Number of iterations and effect of all SNPs examined; Table S2: PRS cross-validation statistics; Table S3: List of all single nucleotide polymorphisms (SNPs) (n = 343) included in the PRS for BMI, sorted by number of times they appeared in the split datasets (largest to smallest).
