# Supplementary material for: Robust Bioinformatics Approaches Result in the First Polygenic Risk Score for BMI in Greek Adults
Source: J Pers Med. 2023 Feb 14;13(2):327. doi: 10.3390/jpm13020327 (PMC9960517; doi:10.3390/jpm13020327)
Supplement: Supplementary file 1 [file jpm-13-00327-s001.zip › Supplementary_figures.pdf]

Supplementary Figures

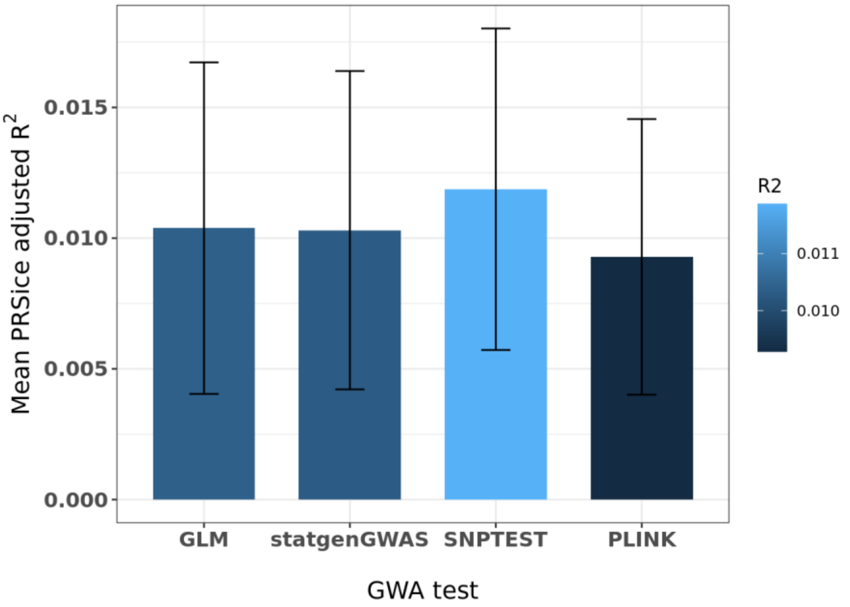

Figure S1: Mean PRSice2 PRS R^2 +/- standard deviation, for each performed summary statistics derivation method across 100 PRS extraction iterations. Higher PRS R^2s are also depicted by lighter blue.

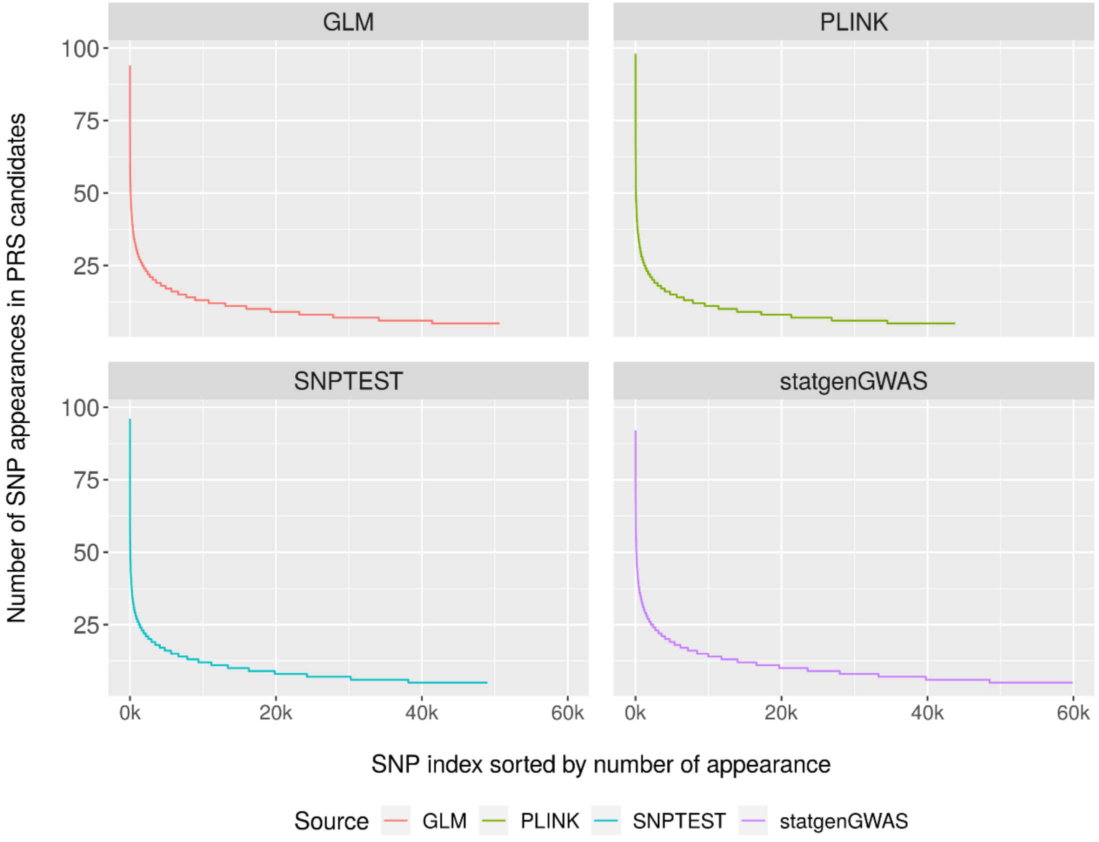

Figure S2: Stability of the PRS candidates over 100 PRS extraction iterations as described in the main text. The vertical axis depicts the number of appearances of each SNP indexed at the horizontal axis, sorted from highest number of appearances to lowest. While a small number of SNPs appears in the majority of iterations, there are many SNPs contributing to the phenotype variability (as measured by their effect and statistical significance) that appear less frequently.

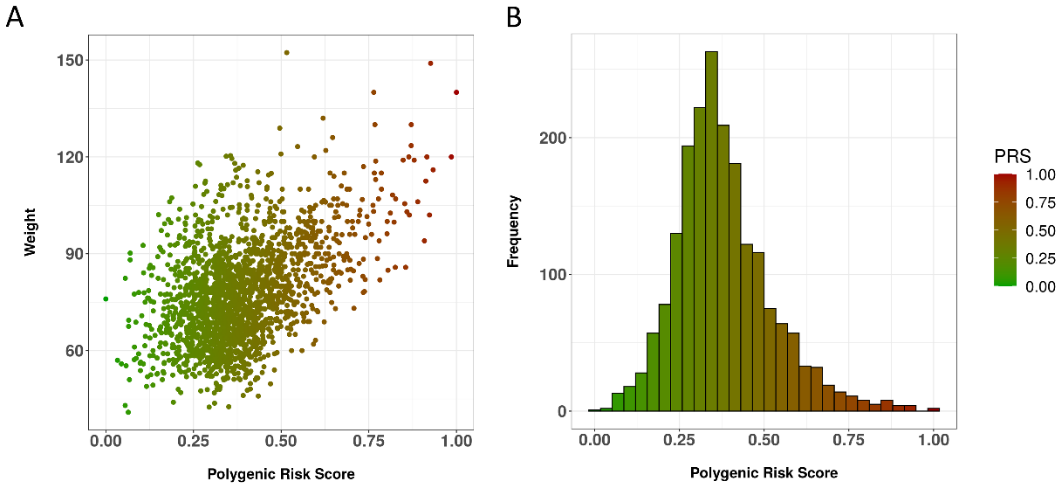

Figure S3: Correlation of the 343-SNP PRS for BMI with the weight phenotype and PRS distribution. A. The weight phenotype across the merged dataset is plotted against the min-max normalized PRS value for each individual. B. Histogram depicting the min-max normalized PRS distribution for all individuals in the merged dataset.
