# Supplementary material for: Robust Bioinformatics Approaches Result in the First Polygenic Risk Score for BMI in Greek Adults
Source: J Pers Med. 2023 Feb 14;13(2):327. doi: 10.3390/jpm13020327 (PMC9960517; doi:10.3390/jpm13020327)
Supplement: Supplementary file 1 [file jpm-13-00327-s001.zip › Supplementary_Table_S2.pdf]

Supplementary Table S2: PRS cross-validation statistics. LO: Leave-Out, Full R^2: The regression R^2 of the full model including the PRS, Reduced R^2: The regression R^2 of the reduced model, excluding the PRS, PRS p-value: The statistical significance of the PRS covariate in the model, t-test p-value: The significance outcome when comparing the mean Reduced R^2 and the mean Full R^2 distributions using the Student's t-test.

LO %	Full R2	Reduced R2	PRS R2	PRS p-value	t-test p-value
5%	0.4301 ± 0.0061	0.1045 ± 0.0028	0.3257 ± 0.0063	8.7378e-181	2.8558e-22
10%	0.4291 ± 0.0056	0.1053 ± 0.0036	0.3237 ± 0.0054	7.3454e-170	3.3106e-26
15%	0.4253 ± 0.0081	0.1031 ± 0.0038	0.3222 ± 0.0098	9.4516e-155	7.6424e-21
20%	0.4265 ± 0.0072	0.1036 ± 0.0053	0.3229 ± 0.0060	4.1215e-147	1.4425e-25
25%	0.4305 ± 0.0146	0.1036 ± 0.0075	0.3269 ± 0.0149	2.2041e-136	2.2658e-18
30%	0.4312 ± 0.0096	0.1033 ± 0.0064	0.3279 ± 0.0114	9.9810e-130	5.4345e-23
35%	0.4285 ± 0.0132	0.1027 ± 0.0120	0.3259 ± 0.0094	4.1673e-120	4.5856e-22
40%	0.4248 ± 0.0188	0.1039 ± 0.0136	0.3209 ± 0.0161	6.4365e-103	1.0911e-18
45%	0.4334 ± 0.0193	0.1055 ± 0.0140	0.3279 ± 0.0153	2.0854e-100	1.0376e-18
50%	0.4218 ± 0.0211	0.1059 ± 0.0115	0.3159 ± 0.0190	4.0321e-87	2.5149e-16
