# Supplementary material for: Robust Bioinformatics Approaches Result in the First Polygenic Risk Score for BMI in Greek Adults
Source: J Pers Med. 2023 Feb 14;13(2):327. doi: 10.3390/jpm13020327 (PMC9960517; doi:10.3390/jpm13020327)
Supplement: Supplementary file 1 [file jpm-13-00327-s001.zip › Supplementary_Table_S3.pdf]

Supplementary Table S3. List of all Single Nucleotide Polymorphisms (SNPs) (n=343) included in the PRS for BMI, sorted by the Nº of times they appeared in the split datasets (largest to smallest).

SNP	chromosome	position	Nearest Gene	Risk Allele	Reference Allele	beta	OR	Frequency
rs11668205	19	2099250	IZUMO4	G	A	-0.325754	1.385075	96
rs11994887	8	4391719	CSMD1	T	C	-0.262923	1.300727	64
rs9295609	6	23979222	#N/A	G	A	-0.221097	1.247444	62
rs4279903	1	205439769	#N/A	G	A	0.198356	0.820078	61
rs16883347	5	9667798	LINC02112	A	C	0.232483	0.792563	60
rs1332010	6	38996875	DNAH8	T	C	0.197648	0.820659	60
rs2108929	7	13146222	#N/A	C	T	-0.281552	1.325184	60
rs12588521	14	30475398	#N/A	A	C	-0.290499	1.337095	60
rs1499951	3	115195109	#N/A	C	T	-0.28408	1.32854	58
rs9848915	3	167160291	SERPINI2	G	A	-0.272895	1.313763	58
rs1213402	5	111112309	NREP	T	C	-0.277976	1.320455	58
rs7407266	18	6568765	#N/A	T	C	0.162551	0.849973	58
rs1461920	1	226794525	STUM	A	G	-0.144307	1.155238	57
rs2691505	6	71310131	#N/A	A	G	0.150908	0.859927	57
rs12941504	17	72478313	CD300A	T	C	-0.213346	1.237812	57
rs4861680	4	186721885	SORBS2	G	A	0.156324	0.855282	56
rs113990664	5	71376237	#N/A	G	A	-0.163085	1.177137	56
rs7122189	11	24407108	#N/A	C	T	-0.219925	1.245984	56
rs7176478	15	66534231	MEGF11	G	A	0.21009	0.810511	56
rs962682	16	84846251	#N/A	A	G	-0.171701	1.187323	56
rs9303353	17	52728276	#N/A	T	G	0.140039	0.869324	56
rs9897526	17	42426940	GRN	G	A	0.191341	0.825851	56
rs1056896	4	185677363	ACSL1	T	C	0.185506	0.830684	55
rs12478622	2	204979611	#N/A	G	A	-0.234437	1.264197	54
rs4906842	15	26274261	LINC02346	A	G	-0.173873	1.189905	54
rs1604953	16	78942601	WWOX	G	A	-0.17161	1.187214	54
rs12959396	18	60039309	TNFRSF11A	T	G	0.147374	0.862971	54
rs12145632	1	247393142	#N/A	T	C	-0.182569	1.200297	53
rs6541160	1	220981358	MTARC1	T	C	-0.209923	1.233583	53

rs756325	1	110478064	#N/A	T	C	0.211103	0.809691	53
rs6827349	4	122980440	#N/A	A	G	0.14465	0.865325	53
rs983585	7	107656377	#N/A	T	C	-0.169588	1.184817	53
rs55906926	3	143159873	SLC9A9	A	G	0.283481	0.753158	52
rs1563556	5	174770776	#N/A	G	A	-0.150676	1.16262	52
rs10500800	11	13929427	#N/A	G	T	-0.230429	1.259141	52
rs56115087	17	71660771	#N/A	A	G	-0.222391	1.249059	52
rs13058401	22	29862247	#N/A	G	A	-0.236776	1.267157	52
rs6677968	1	65776702	DNAJC6	G	A	0.144549	0.865412	51
rs989632	5	7823382	ADCY2	A	G	-0.250872	1.285146	51
rs1128250	7	21956405	CDCA7L	G	A	0.164662	0.848181	51
rs7101190	10	71588349	COL13A1	C	T	0.170232	0.843469	51
rs8064152	16	5721431	#N/A	A	C	-0.224151	1.251259	51
rs1673000	19	35575767	HPN-AS1	T	C	-0.215948	1.241038	51
rs12139329	1	193463330	#N/A	T	C	-0.165837	1.180381	50
rs11904043	2	53644605	#N/A	G	A	-0.160222	1.173771	50
rs16838023	2	207053416	GPR1	G	T	0.16384	0.848878	50
rs1109771	6	32187605	#N/A	A	G	0.151916	0.859061	50
rs10901376	10	127085818	#N/A	G	A	0.15818	0.853696	50
rs7925657	11	44287292	ALX4	A	G	0.142327	0.867337	50
rs488248	13	106596719	#N/A	C	T	-0.170482	1.185876	50
rs12909478	15	33829250	RYR3	C	T	0.16306	0.84954	50
rs12052178	19	46114829	EML2	C	T	-0.136768	1.146562	50
rs17455693	1	165917820	#N/A	A	G	-0.168336	1.183334	49
rs6694023	1	62130167	#N/A	A	G	-0.161533	1.175312	49
rs12466395	2	190780698	#N/A	A	G	-0.143846	1.154706	49
rs8016755	14	25111113	#N/A	C	T	-0.132007	1.141116	49
rs78457560	17	13657119	#N/A	G	A	-0.25513	1.29063	49
rs2839644	21	44604272	#N/A	T	C	-0.17819	1.195052	49
rs480039	1	234426478	SLC35F3	G	A	-0.173613	1.189596	48
rs987174	3	5925865	#N/A	A	G	-0.265751	1.30441	48
rs9877544	3	97684754	RIOX2	G	A	-0.135372	1.144963	48

rs4346760	5	150493470	ANXA6	C	A	-0.127959	1.136506	48
rs1345934	7	136772275	LOC349160	A	G	-0.144756	1.155758	48
rs955975	9	85117420	#N/A	G	A	0.165892	0.847138	48
rs3912662	11	92595671	FAT3	G	A	0.158048	0.853808	48
rs4937166	11	126506712	KIRREL3	C	T	-0.170755	1.1862	48
rs9924586	16	59533731	#N/A	C	T	-0.151622	1.16372	48
rs6016500	20	39634488	#N/A	C	T	0.220392	0.802204	48
rs7264181	20	13231342	ISM1	G	A	0.197286	0.820956	48
rs6449263	4	16669406	LDB2	G	A	-0.123672	1.131645	47
rs6881547	5	158946495	#N/A	G	A	-0.174727	1.19092	47
rs6900225	6	9314807	#N/A	G	A	0.189038	0.827755	47
rs2856330	12	11942777	ETV6	C	T	-0.213801	1.238376	47
rs7989395	13	38010367	#N/A	A	C	-0.172584	1.188372	47
rs7279498	21	42253571	#N/A	G	A	-0.169416	1.184613	47
rs11121141	1	8294662	#N/A	G	A	-0.166324	1.180956	46
rs2807854	1	221029841	#N/A	T	C	-0.13816	1.148159	46
rs237108	6	79415015	#N/A	C	T	-0.161081	1.17478	46
rs2392445	7	36547667	#N/A	A	G	-0.211141	1.235086	46
rs7943949	11	40840931	LRRC4C	T	C	-0.200311	1.221783	46
rs11106815	12	93428691	LOC643339	G	A	-0.190596	1.209971	46
rs575478	12	114166291	#N/A	C	T	-0.257003	1.293049	46
rs2288061	16	76169731	#N/A	G	A	-0.177761	1.19454	46
rs9897341	17	9123894	NTN1	T	C	-0.174271	1.190378	46
rs4599028	19	53970581	#N/A	A	G	-0.18578	1.204157	46
rs6510364	19	33671172	#N/A	C	T	-0.120742	1.128334	46
rs1382425	4	189541446	#N/A	A	C	-0.209858	1.233502	45
rs284729	5	35457590	#N/A	A	G	0.197514	0.820768	45
rs4867856	5	176144471	#N/A	T	C	-0.148169	1.159709	45
rs540375	5	152878727	GRIA1	G	A	-0.138013	1.147991	45
rs233958	16	4340305	#N/A	C	T	-0.146388	1.157646	45
rs17008392	3	71291620	#N/A	A	G	0.240232	0.786446	44
rs13357315	5	56809263	#N/A	T	C	-0.193457	1.213437	44

rs901285	11	12160054	MICAL2	T	C	-0.238964	1.269933	44
rs7144237	14	80897656	DIO2-AS1	A	G	-0.137906	1.147868	44
rs17776995	16	49447822	#N/A	G	A	-0.209867	1.233514	44
rs5765681	22	46259060	#N/A	T	C	-0.167349	1.182166	44
rs11121937	1	12625844	#N/A	T	C	0.13541	0.873358	43
rs16846210	2	212322255	ERBB4	C	T	-0.165111	1.179524	43
rs1357134	3	186979768	MASP1	A	G	-0.136223	1.145937	43
rs2309942	4	184839829	STOX2	G	A	0.138368	0.870778	43
rs13182748	5	5915186	#N/A	C	A	0.156118	0.855458	43
rs697505	6	158816875	TULP4	T	G	-0.175773	1.192167	43
rs6981400	8	23104612	CHMP7	T	C	-0.16746	1.182298	43
rs13297661	9	132926080	#N/A	G	A	0.130994	0.877223	43
rs2852786	11	61514085	DAGLA	C	T	0.145421	0.864659	43
rs3808986	11	129910584	#N/A	C	A	-0.231172	1.260076	43
rs3741565	12	130557260	#N/A	A	G	-0.182105	1.19974	43
rs1959169	14	33473192	NPAS3	C	T	-0.212534	1.236808	43
rs1860304	16	5631166	#N/A	A	G	-0.170029	1.185339	43
rs4807505	19	3750869	APBA3	G	A	0.171137	0.842706	43
rs4925403	22	49070719	TAFA5	G	A	-0.203892	1.226165	43
rs2758615	1	156158367	#N/A	T	C	0.129133	0.878857	42
rs11689662	2	154003392	#N/A	G	A	-0.18104	1.198463	42
rs12614570	2	228651252	#N/A	G	T	0.133843	0.874727	42
rs4852508	2	79906739	CTNNA2	C	T	-0.197861	1.218793	42
rs6730873	2	216346048	#N/A	C	T	0.129299	0.878711	42
rs10935254	3	137254071	#N/A	A	G	-0.150471	1.162382	42
rs7430102	3	54537866	CACNA2D3	G	A	0.185439	0.830739	42
rs9882796	3	189330551	#N/A	G	A	0.147893	0.862523	42
rs6822346	4	190065197	#N/A	G	A	0.138813	0.870391	42
rs7694518	4	7459344	SORCS2	G	A	0.180304	0.835017	42
rs2281144	6	167091844	RPS6KA2	A	G	-0.190103	1.209374	42
rs1420123	7	29647662	#N/A	T	C	0.155306	0.856153	42
rs4979009	9	114347774	PTGR1	A	G	-0.142361	1.152993	42

rs7077407	10	8140574	#N/A	A	G	0.120968	0.886062	42
rs7104900	11	64303696	#N/A	G	A	0.153867	0.857386	42
rs11608342	12	67406881	#N/A	A	G	-0.176385	1.192898	42
rs59038615	12	82253603	#N/A	C	T	-0.17599	1.192427	42
rs78036224	12	39279534	CPNE8	G	T	0.248957	0.779613	42
rs586379	13	27029720	#N/A	T	C	0.179712	0.835511	42
rs11157000	14	38920795	#N/A	G	T	-0.135777	1.145426	42
rs2955742	15	76446132	TMEM266	G	A	-0.191082	1.210559	42
rs12931774	16	6965041	#N/A	G	A	-0.183571	1.2015	42
rs4806908	19	1004823	GRIN3B	G	A	0.134689	0.873987	42
rs7256086	19	327182	MIER2	C	T	-0.20064	1.222184	42
rs8111919	19	54128771	#N/A	G	A	-0.171	1.186491	42
rs11687151	2	87949635	#N/A	C	T	-0.18503	1.203254	41
rs12466549	2	192615315	#N/A	G	A	-0.135485	1.145091	41
rs4341972	2	69042142	ARHGAP25	G	A	0.138324	0.870816	41
rs483394	2	14137805	#N/A	C	T	0.156618	0.85503	41
rs62107643	2	3882074	#N/A	G	A	-0.244019	1.276368	41
rs9878545	3	171621021	#N/A	T	C	-0.120873	1.128481	41
rs1400871	5	124678629	#N/A	C	A	-0.144597	1.155573	41
rs3101186	5	71409569	MAP1B	A	C	0.198388	0.820052	41
rs440583	5	80322231	RASGRF2	T	C	-0.169856	1.185134	41
rs12192629	6	8426587	SLC35B3	G	T	-0.159928	1.173426	41
rs1556675	6	141737996	#N/A	T	C	0.162115	0.850343	41
rs2472802	6	14767419	#N/A	G	A	-0.169044	1.184172	41
rs6455019	6	65668333	EYS	T	C	-0.180085	1.197319	41
rs6906804	6	1490582	#N/A	G	A	0.150242	0.860499	41
rs6918155	6	167145388	RPS6KA2	G	A	0.16043	0.851778	41
rs12681882	8	103578377	#N/A	G	A	-0.178524	1.195452	41
rs36107033	8	37556987	ZNF703	C	T	-0.247376	1.28066	41
rs10959370	9	10923969	#N/A	G	T	0.249514	0.779179	41
rs10508741	10	29252309	#N/A	T	G	-0.146386	1.157642	41
rs11259736	10	15595361	ITGA8	G	A	-0.167693	1.182573	41

rs2813458	10	1574443	ADARB2-AS1	C	T	-0.175466	1.191802	41
rs11224017	11	134655690	#N/A	T	C	0.150652	0.860147	41
rs12581737	12	63125469	PPM1H	T	G	-0.196294	1.216885	41
rs879681	13	106800033	#N/A	G	A	0.197196	0.821029	41
rs8003359	14	57126170	#N/A	T	C	-0.181305	1.198781	41
rs8013199	14	23247486	SLC7A7	T	C	-0.181414	1.198912	41
rs1015310	15	102110442	#N/A	G	T	-0.132038	1.141152	41
rs11648289	16	7773250	#N/A	G	T	-0.129338	1.138075	41
rs1561699	18	47996600	#N/A	C	T	-0.162987	1.177021	41
rs9304414	18	49515554	#N/A	T	G	-0.152022	1.164185	41
rs2076408	20	2313059	TGM3	T	G	-0.161035	1.174727	41
rs817362	20	62559964	DNAJC5	G	A	-0.22142	1.247847	41
rs857138	1	57191924	FYB2	C	T	-0.156906	1.169885	40
rs16838452	2	202798759	#N/A	C	A	-0.181695	1.199249	40
rs1354350	3	113960449	#N/A	T	G	-0.130177	1.13903	40
rs1405406	3	73946124	#N/A	G	A	-0.180443	1.197747	40
rs2863384	3	166085645	#N/A	C	T	0.180731	0.83466	40
rs17237942	4	105425576	#N/A	A	G	-0.146612	1.157905	40
rs2503815	6	5391268	FARS2	G	A	-0.185625	1.203971	40
rs17171046	7	37477863	ELMO1	C	T	-0.177167	1.193831	40
rs6989157	8	96320757	C8orf37-AS1	G	A	-0.189935	1.209171	40
rs9325887	10	48461083	#N/A	A	G	0.168659	0.844797	40
rs342174	12	63065145	PPM1H	G	A	-0.182831	1.200611	40
rs10148212	14	67095613	GPHN	A	C	-0.192537	1.212322	40
rs1822809	15	95798343	#N/A	G	A	-0.16178	1.175601	40
rs11866097	16	55104462	#N/A	G	A	-0.197333	1.21815	40
rs2589141	17	78843139	RPTOR	C	T	-0.148067	1.15959	40
rs4790904	17	64779154	PRKCA	T	C	-0.138626	1.148695	40
rs55667536	17	39293446	#N/A	A	G	-0.331439	1.392971	40
rs1106978	20	41678428	PTPRT	G	A	-0.14801	1.159525	40
rs6007778	22	48392923	#N/A	G	A	-0.200854	1.222446	40
rs11811436	1	97940231	DPYD	T	C	0.174909	0.839533	39

rs147762374	1	106873477	#N/A	G	A	-0.160322	1.173889	39
rs2376100	2	197833530	#N/A	T	C	0.270528	0.762976	39
rs6712770	2	164106998	#N/A	A	C	-0.161215	1.174937	39
rs1163377	3	108937719	#N/A	G	A	-0.1455	1.156618	39
rs11922267	3	65401156	MAGI1	C	T	-0.176977	1.193604	39
rs2649188	3	65780102	MAGI1	A	C	0.122599	0.884619	39
rs7620686	3	26413729	#N/A	T	C	-0.116506	1.123565	39
rs10076573	5	2094148	#N/A	T	C	0.165889	0.847141	39
rs10477436	5	108421424	FER	A	C	-0.129262	1.137988	39
rs4388209	5	125758373	GRAMD2B	T	C	0.113824	0.892415	39
rs6925019	6	1588714	#N/A	G	A	-0.179622	1.196765	39
rs2710804	7	36084529	#N/A	T	C	-0.135608	1.145233	39
rs4724298	7	44320434	CAMK2B	C	T	-0.179291	1.196369	39
rs2173537	8	129047325	PVT1	G	A	-0.180237	1.197501	39
rs1556146	9	132022478	#N/A	T	C	-0.198734	1.219857	39
rs16907144	9	93852382	#N/A	G	A	0.158523	0.853403	39
rs824003	9	113972955	#N/A	T	C	-0.162957	1.176986	39
rs12772906	10	62804925	#N/A	A	G	-0.184202	1.202258	39
rs12417297	11	128185154	#N/A	C	T	-0.232895	1.262248	39
rs10492094	12	5478148	#N/A	G	T	-0.125703	1.133945	39
rs1544686	12	107579464	#N/A	G	T	-0.184251	1.202317	39
rs17091684	12	43076137	#N/A	C	A	-0.169665	1.184908	39
rs1170994	13	36581177	DCLK1	C	T	0.161757	0.850648	39
rs9533784	13	44761837	#N/A	G	A	-0.182495	1.200208	39
rs10146690	14	79890456	NRXN3	G	A	-0.145193	1.156263	39
rs11621403	14	53877638	#N/A	A	G	0.203013	0.816268	39
rs1791159	18	29136463	DSG2-AS1	T	C	-0.134932	1.144459	39
rs275339	20	57988701	#N/A	C	T	0.17152	0.842384	39
rs4816499	21	36293381	RUNX1	A	C	-0.256245	1.292069	39
rs10458443	1	242712951	#N/A	T	C	0.157657	0.854143	38
rs285461	1	165497692	LRRC52-AS1	T	C	-0.221145	1.247505	38
rs4559489	1	240698138	GREM2	C	T	-0.232214	1.26139	38

rs12473883	2	236559912	AGAP1	G	T	-0.163999	1.178213	38
rs13405786	2	70844394	#N/A	T	C	-0.190585	1.209958	38
rs4253427	4	187209225	F11-AS1	G	A	0.127626	0.880183	38
rs11759402	6	42723809	#N/A	C	T	-0.154175	1.166695	38
rs1248465	7	129179844	#N/A	C	T	-0.118936	1.126298	38
rs2251188	7	6704332	#N/A	A	G	0.138076	0.871032	38
rs6973044	7	107416602	SLC26A3	T	C	-0.228111	1.256225	38
rs11780206	8	59385164	#N/A	T	C	-0.204419	1.226812	38
rs11783343	8	62673021	#N/A	T	C	-0.178607	1.195551	38
rs12246329	10	17059462	CUBN	C	T	-0.148457	1.160043	38
rs4751284	10	132653619	#N/A	G	A	0.116341	0.890172	38
rs1032151	11	12219217	MICAL2	G	T	-0.1486	1.160209	38
rs11602776	11	15448704	#N/A	A	G	0.170025	0.843643	38
rs11106473	12	92627100	#N/A	T	C	-0.185816	1.2042	38
rs9562038	13	96802616	HS6ST3	A	G	-0.166981	1.181732	38
rs34361729	15	56387142	RFX7	G	A	-0.211004	1.234918	38
rs4794785	17	36810197	#N/A	A	G	0.226712	0.797151	38
rs8464	17	64806716	PRKCA	C	A	-0.175982	1.192417	38
rs2422840	20	3076384	#N/A	A	G	-0.170721	1.18616	38
rs6065084	20	59862269	CDH4	G	A	0.157302	0.854446	38
rs1786412	21	21849932	#N/A	T	C	-0.144162	1.155071	38
rs7275495	21	33533452	#N/A	T	C	-0.159611	1.173054	38
rs1287949	1	226703467	#N/A	C	T	-0.135919	1.145589	37
rs2297563	1	109241669	PRPF38B	G	A	-0.138652	1.148725	37
rs3849346	2	163502721	KCNH7	C	T	-0.191176	1.210672	37
rs7589592	2	2712943	#N/A	T	C	0.113915	0.892334	37
rs10939667	4	16531086	LDB2	G	A	0.143542	0.866285	37
rs72696876	4	171635905	#N/A	A	G	0.17133	0.842543	37
rs11752561	6	128187461	THEMIS	A	G	0.178228	0.836752	37
rs2382394	9	13227063	MPDZ	T	C	-0.295083	1.343238	37
rs10508561	10	18734235	CACNB2	G	A	0.155748	0.855775	37
rs4143630	10	5063944	#N/A	C	T	-0.125875	1.134141	37

rs4261213	10	58261332	#N/A	G	A	0.121382	0.885696	37
rs10765861	11	11525424	GALNT18	A	G	-0.123483	1.131431	37
rs2581925	11	57207414	#N/A	G	A	0.135819	0.873	37
rs1446427	12	118153788	KSR2	G	A	0.145306	0.864758	37
rs2274442	14	69787749	GALNT16	T	C	-0.222228	1.248856	37
rs7144194	14	103495963	CDC42BPB	A	G	-0.131499	1.140536	37
rs1476078	15	91736401	SV2B	G	A	-0.207155	1.230173	37
rs2880974	15	86415200	#N/A	G	T	-0.143929	1.154802	37
rs12928792	16	60321890	#N/A	G	A	-0.11811	1.125368	37
rs9807305	18	59634276	#N/A	C	T	0.176512	0.838188	37
rs12118086	1	101732914	#N/A	G	A	0.181836	0.833738	36
rs1570818	1	29804613	#N/A	A	C	0.161503	0.850864	36
rs17366341	1	163834280	#N/A	A	G	-0.162322	1.176239	36
rs2485662	1	156083468	#N/A	T	C	0.116015	0.890461	36
rs784612	1	40122939	#N/A	T	C	-0.173568	1.189541	36
rs4850836	2	199122020	#N/A	C	A	-0.146091	1.157302	36
rs17009383	3	21771769	ZNF385D	A	G	0.168139	0.845236	36
rs7623055	3	7510891	GRM7	G	T	0.123122	0.884156	36
rs9847987	3	59418533	#N/A	C	T	0.26275	0.768934	36
rs1491386	4	23663069	#N/A	A	C	0.208738	0.811608	36
rs7732443	5	166290093	#N/A	C	T	0.1964	0.821683	36
rs2459110	6	152450763	SYNE1	C	A	-0.123803	1.131793	36
rs9363725	6	67931025	#N/A	G	A	0.277937	0.757344	36
rs9377661	6	105091850	#N/A	C	T	0.189219	0.827605	36
rs12673506	7	29421786	CHN2	G	A	-0.185004	1.203223	36
rs4718965	7	70040448	AUTS2	C	T	-0.190496	1.20985	36
rs4729041	7	92063500	#N/A	G	T	-0.18693	1.205543	36
rs10101912	8	88218888	CNBD1	C	T	-0.217885	1.243444	36
rs3913641	8	140342525	#N/A	C	T	-0.127474	1.135955	36
rs12337816	9	117125236	AKNA	T	C	-0.222317	1.248967	36
rs17662327	9	4967587	#N/A	T	C	0.14715	0.863165	36
rs7861175	9	113857337	#N/A	T	C	0.140404	0.869007	36

rs17106110	10	125601307	CPXM2	C	T	-0.139741	1.149975	36
rs10899014	11	74325109	POLD3	A	G	-0.299626	1.349355	36
rs2302688	12	15673103	PTPRO	C	T	0.13391	0.874669	36
rs7295237	12	71016832	PTPRB	T	C	-0.252748	1.287558	36
rs10851193	13	107335929	#N/A	T	C	0.105358	0.900002	36
rs1617356	14	43194459	#N/A	C	T	-0.131898	1.140992	36
rs17126387	14	90301314	EFCAB11	A	C	0.223227	0.799933	36
rs7149564	14	23912361	#N/A	T	C	-0.261682	1.299113	36
rs8009629	14	33379559	#N/A	G	A	-0.130628	1.139543	36
rs13335201	16	50912221	#N/A	G	A	-0.171548	1.187141	36
rs1350888	16	82784943	CDH13	C	T	0.11881	0.887977	36
rs12948545	17	30867317	MYO1D	C	T	-0.139738	1.149973	36
rs218685	17	6623172	#N/A	A	G	0.142751	0.86697	36
rs1790692	18	28644751	DSC2	G	A	-0.16607	1.180656	36
rs7241127	18	6651210	#N/A	T	C	-0.140085	1.150371	36
rs8088662	18	60928341	BCL2	T	C	0.212307	0.808717	36
rs1010304	20	40101647	CHD6	A	G	-0.28657	1.331852	36
rs1016071	20	40931328	PTPRT	T	C	-0.123569	1.131528	36
rs6019784	20	48002575	KCNB1	C	T	0.137512	0.871524	36
rs6084802	20	511356	CSNK2A1	C	A	-0.13511	1.144663	36
rs1808973	1	112487834	KCND3	C	T	-0.119334	1.126746	35
rs3820383	1	202197551	LGR6	C	A	0.171723	0.842213	35
rs4658879	1	231742575	LINC00582	T	C	-0.174403	1.190535	35
rs6436132	2	220160644	PTPRN	G	T	0.137995	0.871103	35
rs12485826	3	71342132	#N/A	A	G	-0.16939	1.184582	35
rs75838234	3	7534370	GRM7	A	G	-0.184033	1.202056	35
rs11726275	4	185348916	IRF2	A	G	-0.132627	1.141824	35
rs13128106	4	25325264	ZCCHC4	C	T	-0.237812	1.26847	35
rs6810740	4	17294785	#N/A	T	C	-0.159123	1.172483	35
rs6821900	4	158574310	#N/A	A	C	-0.188469	1.2074	35
rs2567819	5	121064897	#N/A	C	A	0.195192	0.822676	35
rs3853241	5	166379772	#N/A	C	T	-0.170603	1.186019	35

rs10252228	7	34940039	#N/A	A	G	0.120631	0.886361	35
rs17158276	7	29648077	#N/A	T	C	-0.178448	1.195361	35
rs73371641	8	142864765	#N/A	A	G	-0.251769	1.286298	35
rs2384792	9	138324679	#N/A	C	T	-0.129733	1.138525	35
rs4295727	9	112578906	PALM2AKAP2	G	A	-0.141765	1.152306	35
rs9696313	9	136406725	ADAMTSL2	A	G	-0.123854	1.13185	35
rs10824306	10	77160682	ZNF503	C	T	-0.144774	1.155778	35
rs11013723	10	18632601	CACNB2	A	G	0.205084	0.814579	35
rs12221258	10	107582259	#N/A	T	C	-0.149856	1.161666	35
rs2265958	10	10141681	#N/A	C	T	-0.182243	1.199906	35
rs7894073	10	28648001	#N/A	C	T	-0.256118	1.291906	35
rs12290206	11	107761113	#N/A	A	G	-0.153478	1.165882	35
rs4938044	11	113584177	#N/A	G	A	-0.204375	1.226758	35
rs12584740	13	98315257	#N/A	A	G	-0.140091	1.150379	35
rs714668	13	105019361	#N/A	C	A	0.237791	0.788368	35
rs73168114	13	22716081	#N/A	A	C	-0.221493	1.247939	35
rs7323430	13	65285662	#N/A	G	A	0.150152	0.860577	35
rs10131354	14	25499521	STXBP6	T	G	-0.183645	1.201589	35
rs2842344	14	68976971	RAD51B	C	T	-0.199264	1.220504	35
rs71415931	14	30683170	#N/A	T	C	-0.203381	1.22554	35
rs7499658	16	14430442	#N/A	C	T	0.15436	0.856963	35
rs2332264	17	52830577	#N/A	T	C	0.132596	0.875819	35
rs2850929	18	75068081	#N/A	G	A	0.157779	0.854039	35
rs755719	18	56877489	#N/A	T	C	0.111622	0.894382	35
rs762418	21	45610893	#N/A	C	T	0.142348	0.867319	35
rs5761487	22	26738225	SEZ6L	A	G	0.141173	0.868339	35
